# Investigation of the Perceptions, Attitudes, and Influences Towards Plastic Surgery Among Medical Students in Oman: A Questionnaire-Based Study

**DOI:** 10.7759/cureus.79542

**Published:** 2025-02-24

**Authors:** Abdullah Al Lawati, Firas Al Majarfi, Mohammed M Allouyahi, Musaab Al-Hinaai, Meetham Al Lawati, Sheikhan Al Hashmi, Nawaf Al-Muqaimi

**Affiliations:** 1 College of Medicine and Health Sciences, Sultan Qaboos University, Muscat, OMN; 2 Department of Plastic Surgery, Khoula Hospital, Muscat, OMN; 3 Department of Surgery, Sultan Qaboos University Hospital, Muscat, OMN

**Keywords:** attitudes, influences, medical students, perceptions, plastic and reconstructive surgery

## Abstract

Introduction

Plastic surgery encompasses cosmetic and reconstructive procedures to restore, enhance, or reconstruct body structures. Unlike other specialties, it is not confined to specific anatomical areas, organ systems, or age groups. Despite significant growth in the field, it remains poorly understood by healthcare professionals, medical students, and the public. Misconceptions about the scope and procedures of plastic surgery persist, potentially influencing career choices and referral patterns. This study explores the knowledge, attitudes, and perceptions of medical students at Sultan Qaboos University (SQU), Muscat, Oman, regarding plastic surgery, marking, to the best of our knowledge, the first investigation of this topic in Oman.

Methods

A cross-sectional observational study was conducted using a validated questionnaire to assess plastic surgery awareness among undergraduate medical students at SQU. The sample included 310 medical students, excluding foundation-level students. Statistical analysis was performed using IBM SPSS Statistics for Windows, Version 27 (Released 2020; IBM Corp., Armonk, NY, USA), and ethical approval was obtained from the Medical Research and Ethics Committee at SQU.

Results

The study revealed a high level of awareness among students, with 194 (62.6%) correctly identifying cosmetic surgery as a subset of plastic surgery. However, misconceptions persisted, with 65 (20.9%) labeling plastic surgery and cosmetic surgery as separate entities, 19 (6.1%) choosing plastic and cosmetic surgery as the same thing, and 32 (10.3%) uncertain. The internet was the primary information source for 217 (70.0%) participants, and Instagram for 182 (58.7%) participants. Only 26 (8.4%) participants selected plastic surgery as a future career choice, while 157 (50.6%) preferred other specialties. Age and academic level significantly influenced decision-making.

Conclusion

While SQU medical students demonstrated moderate awareness of plastic surgery, gaps in knowledge remain. Increased exposure through curricula and clinical rotations could enhance understanding and interest in this specialty, addressing misconceptions and improving patient care outcomes.

## Introduction

Plastic surgery is a specialty that includes restoration, reconstruction, and enhancement of body function and the appearance of structures that are missing, defective, damaged, or misshapen. It consists of two parts: cosmetic and reconstructive surgery [1]. Unlike other medical specialties, plastic surgery is not defined by an anatomic area (obstetrics/gynecology (OB/GYN), ear, nose, and throat (ENT), and thoracic surgery), organ system (gastroenterology and urology), or patient age group (pediatrics, adolescent medicine, and geriatrics) [2]. According to the American Society of Plastic Surgeons, approximately 17.1 million cosmetic procedures and 5.8 million reconstructive procedures were performed in 2016 alone. This represents a surge in cosmetic procedures, performed by 132% since 2000 [3]. Despite this growth, plastic surgery is still misunderstood as a medical specialty by various groups, including primary care physicians, nurses, medical students, and the public [4]. This misunderstanding may stem from the limited exposure to plastic surgery during medical school training, as well as the overlap of procedures from other specialties. Such misunderstanding also mainly concerns the scope of plastic surgery and what procedures fall under it [1,5].

A study conducted in India found that plastic surgery is poorly understood in the medical community, as 12% of the sample believed that plastic and cosmetic surgeries were the same [1]. A study conducted in Saudi Arabia showed a significant relationship between the knowledge of plastic surgery and cultural factors. The study found that people's primary source of information regarding the specialty of plastic surgery was information learned through conversations with friends and family, followed by social media [6]. Other studies found that television is key in shaping the public's understanding of plastic surgery [7,8].

Regarding the knowledge and awareness of medical students on plastic surgery, Mortada et al. concluded that medical students are not adequately knowledgeable about plastic surgery and that early exposure to this specialty may enhance this awareness [9]. As these medical students will eventually become practicing physicians, their misunderstandings about plastic surgery may negatively affect the specialty by altering patient referral patterns. In addition, they need more knowledge and exposure to the specialty to consider it a future career choice [4]. Hence, this study aims to measure the attitudes, influences, and perceptions of medical students at Sultan Qaboos University (SQU), Muscat, Oman, toward plastic surgery. Medical students were selected as they represent future healthcare professionals whose knowledge and perceptions can influence patient counseling, referrals, and career choices. Additionally, as they have foundational medical knowledge, they provide a perspective distinct from the general public while still being in the early stages of professional training. To the best of our knowledge, this is the first study to be conducted in Oman on this topic.

## Materials and methods

A cross-sectional observational study was conducted using a validated questionnaire previously utilized in other studies, with permission granted for its use. The questionnaire was slightly adapted to fit our specific context [9]. The study aimed to assess the awareness of plastic surgery among medical students at SQU. Medical students from SQU were invited to fill out the questionnaire, which was disseminated through Google Forms (Google, Inc., Mountain View, CA, USA). The study description and the consent form were displayed at the beginning of the form. All data were collected using a Microsoft Excel 2018 (Microsoft® Corp., Redmond, WA, USA) spreadsheet.

The questionnaire included a consent page, which ensured that the student had read the consent sheet attached and had given approval to participate in the study. Next, the questionnaire included a demographics page, which collected students' socio-demographic data, including their gender, age, nationality, phase/level of study, and academic grade point average (GPA). Following that, the questionnaire included questions assessing what students know about plastic surgery, how they differentiate plastic surgery from cosmetic surgery, and whether they know why the specialty is called plastic surgery. After that, a section exploring students' fears of getting a scar after plastic surgery and the risks of having plastic or cosmetic surgery was included. Finally, a section assessing whether the students want to be plastic surgeons or not, and where they get their information about plastic surgery, wrapped up the questionnaire.

Statistical analyses were conducted using IBM SPSS Statistics for Windows, Version 27 (Released 2020; IBM Corp., Armonk, NY, USA). Statistical tests were used to examine the prevalence of awareness toward plastic surgery among medical students at SQU. A total of 805 students are enrolled at the College of Medicine and Health Sciences at SQU. Thus, the targeted sample size for this study was 261 respondents, calculated using the population mean formula with a 95% confidence level and a 5% margin of error. Undergraduate medical students from all phases at SQU were included, while foundation students, who had not yet been exposed to medical topics, were excluded. Notably, the study exceeded its target, achieving a total of 310 responses. Approximately 420 students were invited to participate, resulting in 310 responses, yielding a response rate of 74%. Ethical approval was obtained from the Medical Research and Ethics Committee (MREC) at SQU (MREC/3262).

## Results

Data was collected via an online questionnaire, with responses from 310 participants used for the analysis. Of these, 155 were males and 155 were females. The largest group is in Phase 2 (Semesters 3 and 4), comprising 92 (29.7%) participants. The most common GPA range was between 3.0 and 3.49, with 122 (39.4%) participants falling under this category. More than half, specifically 174 participants (56.1%), reported exposure to medically themed television and surgical disciplines, indicating a relatively high level of awareness and the potential influence of media and clinical exposure on their educational and career considerations. Despite this exposure, only a small fraction, 26 (8.4%) participants, had definitively chosen plastic surgery as a career, with a significant portion, 127 (41.0%) participants, still undecided or undetermined. The demographic data of the sample is displayed in Table 1.

**Table 1:** Summary of characteristics and responses of participants

Characteristics and responses	n (%)
Gender	
Female	155 (50)
Male	155 (50)
Educational level	
Phase 1	46 (14.8)
Phase 2 (Sem 1, 2)	53 (17.1)
Phase 2 (Sem 3, 4)	92 (29.7)
Junior clerkship	70 (22.6)
Senior clerkship	49 (18.5)
Academic grade point average	
3.5 - 3.99	58 (18.7)
3.49 - 3	122 (39.4)
2.99 - 2.5	110 (35.5)
<2.5	20 (6.5)
Exposed to medically themed television	
Yes	174 (56.1)
No	136 (43.9)
Exposed to a surgical discipline	
Yes	174 (56.1)
No	136 (43.9)
Made a decision about choosing plastic surgery	
Yes	26 (8.4)
No	157 (50.6)
Undecided/undetermined yet	127 (41.0)

**Figure 1:**
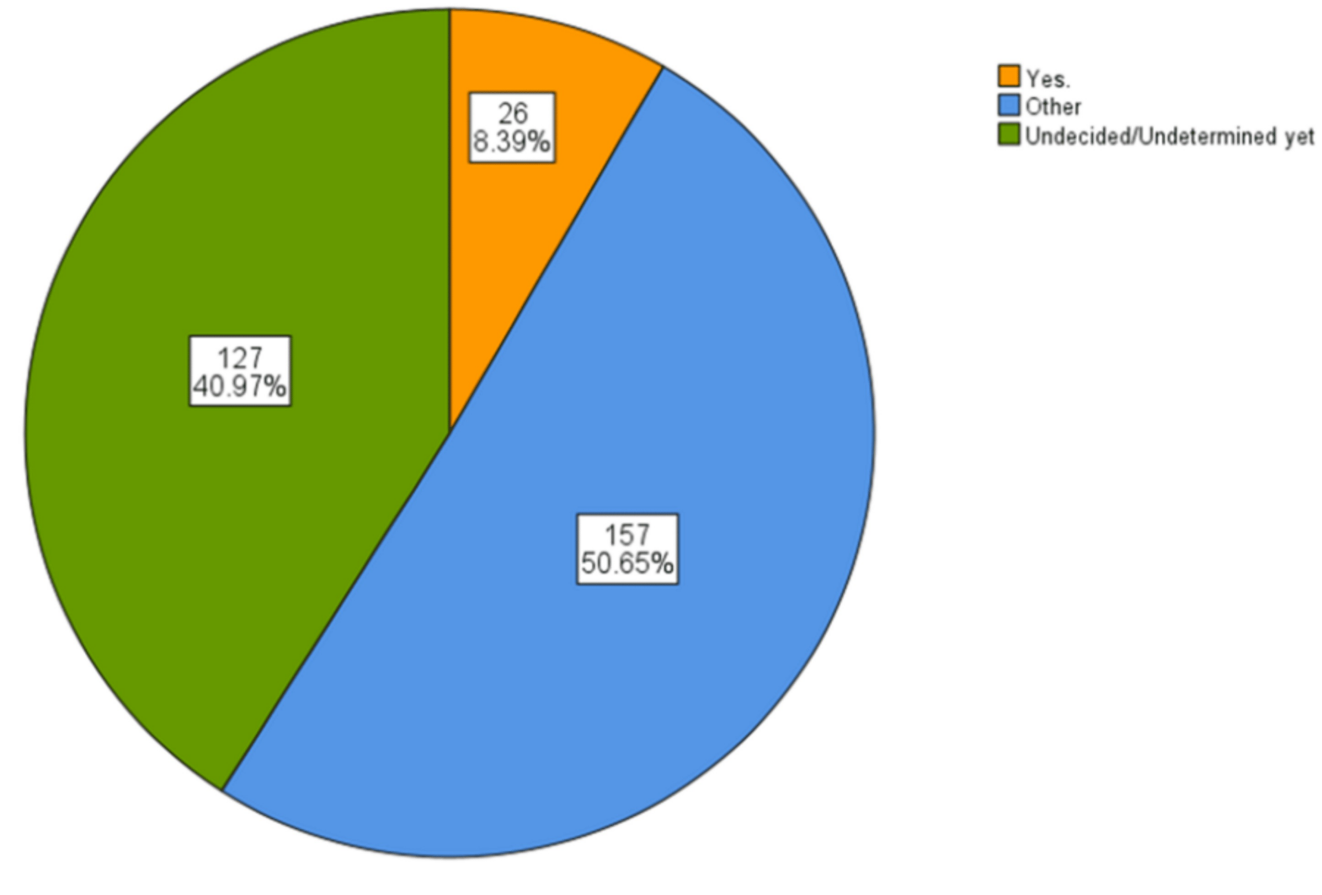
Specialty preferences among participants

As illustrated in Figure 1, the analysis of specialty preferences among the participants reveals a diverse range of interests, with a relatively small proportion expressing a definitive inclination toward plastic surgery. Twenty-six participants, representing 8.4% of the total sample, indicated that their preferred specialty is plastic surgery. Conversely, a more significant portion of the sample - 50.6% or 157 participants, specifically - reported a preference for other specialties outside of plastic surgery. In addition to those who have made firm choices, a significant percentage of participants - 41.0% - remain undecided or undetermined about their specialty preference.

As shown in Table 2, exposure to medically themed television was found to be moderately prevalent among participants, with 56% (n = 174) having such exposure (mean score = 0.56, SD = 0.49), compared to 44% (n = 136) who did not (mean score = 0.44, SD = 0.49). Similarly, exposure to a surgical discipline also showed a parallel trend, with 56% (n = 174, mean score = 0.56, SD = 0.49) reporting exposure and 44% (n = 136, mean score = 0.44, SD = 0.49) not exposed. This pattern indicates that exposure to medically themed television and surgical disciplines may have a comparable influence on participants' considerations regarding specialty choice.

**Table 2:** Achieved scores across various factors

Characteristic	n	Mean score (SD)
Gender		
Female	155	0.50 (0.50)
Male	155	0.50 (0.50)
Exposed to medically themed television		
Yes	174	0.56 (0.49)
No	136	0.44 (0.49)
Exposed to a surgical discipline		
Yes	174	0.56 (0.49)
No	136	0.44 (0.49)
Made a decision about choosing plastic surgery		
Yes	26	0.08 (0.28)
No	157	0.51 (0.50)
Undecided/Undetermined yet	127	0.41 (0.50)
Educational level		
Phase 1	46	0.15 (0.36)
Phase 2 (Sem 1, 2)	53	0.17 (0.38)
Phase 2 (Sem 3, 4)	92	0.30 (0.46)
Junior clerkship	70	0.23 (0.42)
Senior clerkship	49	0.16 (0.36)

Regarding decisions related to choosing plastic surgery as a career, a small proportion (8%, or n = 26) had definitively decided on this specialty (mean score = 0.08, SD = 0.28). In contrast, the majority (51%, or n = 157) had chosen other specialties (mean score = 0.51, SD = 0.50). A considerable portion of participants (41%, or n = 127) remained undecided or undetermined about their specialty preference (mean score = 0.41, SD = 0.50). This distribution suggests that, while interest in plastic surgery exists, most participants have either selected alternative specialties or are still contemplating their choices.

When examining the educational level, the participants were distributed across different stages of medical training. Those in Phase 1 constituted 15% (n = 46, mean score = 0.15, SD = 0.36), Phase 2 (Semesters 1 and 2) accounted for 17% (n = 53, mean score = 0.17, SD = 0.38), and Phase 2 (Semesters 3 and 4) comprised the largest group at 30% (n = 92, mean score = 0.30, SD = 0.46). Participants in the Junior Clerkship stage made up 23% (n = 70, mean score = 0.23, SD = 0.42), while those in the Senior Clerkship represented 16% (n = 49, mean score = 0.16, SD = 0.36). These findings illustrate that specialty preference decisions vary across different educational phases. The high number of undecided participants suggests that students are still exploring their options rather than already committing to a specialty.

Table 3 shows that the study highlights the awareness of medical students toward conditions treated in plastic surgery. The most common conditions that students recognized as treated by plastic surgeons included rhinoplasty (nose jobs) (72.3%, or n = 224), breast reduction or enhancement surgeries (66.1%, or n = 205), and cleft lip and palate (63.5%, or n = 197). Cosmetic procedures such as Botox (61.9%, or n = 192), abdominoplasty (tummy tuck) (43.2%, or n = 134), and liposuction (41.6%, or n = 129) were also frequently noted. Less commonly chosen conditions involved fractures of the hand (19.0%, or n = 59), diabetic foot wounds (16.5%, or n = 51), and tendon injuries of the hand (13.2%, or n = 41).

**Table 3:** Conditions treated in plastic surgery, with frequency referring to the number of students aware of these conditions

Condition	Frequency, n (%)
Eyelid tears and cuts over the face	197 (63.5)
Fractures of the jaw and face	143 (46.1)
Bed sores	36 (11.6)
Diabetic foot wounds	51 (16.5)
Hair transplantation	114 (36.8)
Cleft lip and palate (congenital)	197 (63.5)
Fractures of the hand	59 (19.0)
Injuries to nerves of hand and legs	36 (11.6)
Rhinoplasty (nose job)	224 (72.3)
Finger amputations	78 (25.2)
Burns	193 (62.3)
Congenital anomalies of ear and nose	186 (60.0)
Abdominoplasty (tummy tuck)	134 (43.2)
Botox	192 (61.9)
Liposuction (fat aspiration)	129 (41.6)
Sex change surgery	134 (43.2)
Tendon injuries of hand	41 (13.2)
Breast reduction or enhancement surgeries	205 (66.1)
Breast reconstruction post cancer	196 (63.2)

The study explored various sources influencing participants' knowledge and perceptions of plastic surgery, as shown in Table 4. The most frequently cited source was the internet, with 217 (70.0%) of participants indicating it as a key influence. Instagram was also a significant platform, influencing 182 (58.7%) of participants, while friends were mentioned by 118 participants (38.0%) as a source of information. Traditional media, such as TV, played a role for 97 (31.3%) participants, whereas clinical rotations and teaching sessions were reported by 64 (20.6%) and 67 (21.9%) participants, respectively.

**Table 4:** Sources of information regarding plastic surgery

Source	Frequency, n (%)
Teaching sessions	68 (21.9)
Clinical rotations	64 (20.6)
Personal encounter	67 (21.6)
TV	97 (31.3)
Magazines	31 (10)
Friends	118 (38)
Workplace	41 (13.2)
Internet	217 (70)
Personal experience	35 (11.3)
Instagram	182 (58.7)
Snapchat	56 (18.0)
Twitter	68 (21.9)
Facebook	2 (0.6)

Table 5 examined the correlation between various variables and their influence on participants' decision-making in plastic surgery. Age showed a strong positive correlation (r = 0.758) with a highly significant p-value (<0.001), indicating that age is a significant factor affecting participants' decisions. The educational level had a weak positive correlation (r = 0.190) but remained statistically significant (p < 0.001), suggesting a minor influence on decision-making. In contrast, the academic GPA showed a moderate positive correlation (r = 0.310); however, the lack of a p-value suggests that further analysis is needed to determine its statistical significance. Overall, age appears to be the most influential factor, followed by educational level and GPA, as shown in Table 5.

**Table 5:** Spearman correlation test for the correlation between achieved score and age, educational level, and academic grade point average (N = 310)

Variable	Correlation coefficient (r)	p-value
Age	0.758	<0.001
Educational level	0.190	<0.001
Academic grade point average	0.310	-

**Figure 2:**
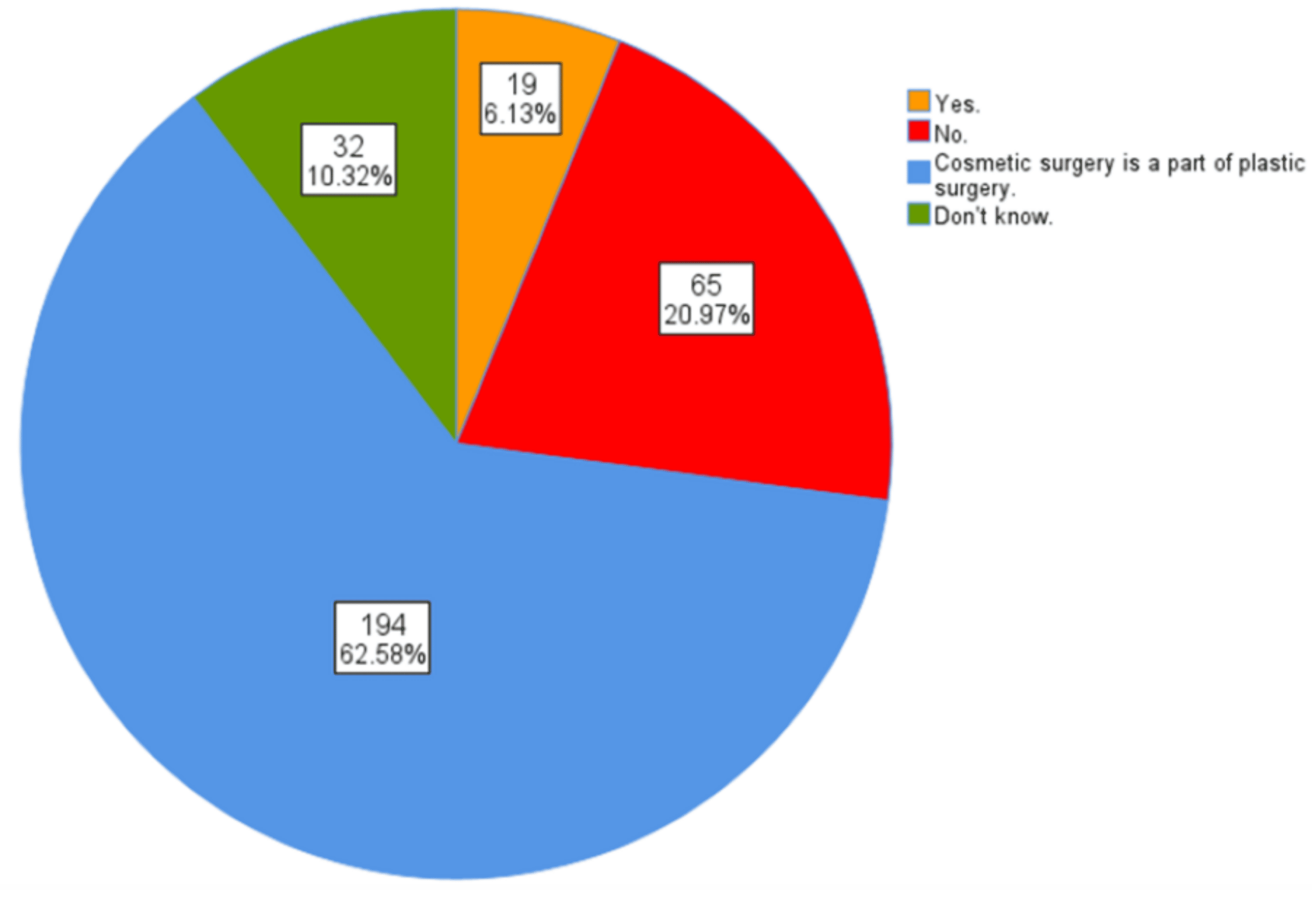
Distribution of public responses regarding the relationship between cosmetic surgery and plastic surgery

This study also highlighted medical students' understanding of the terms "plastic surgery" and "cosmetic surgery," aiming to assess their comprehension of whether cosmetic surgery is a subset of plastic surgery, whether the two fields are the same and the terms are used interchangeably, or if the fields are separate and unrelated. Figure 2 demonstrates the distribution of responses regarding the relationship between cosmetic surgery and plastic surgery, and whether they are considered one part or not. The majority of the participants, 195 (62.9%), recognized that cosmetic surgery is a part of plastic surgery. However, a significant number of participants, 65 (21.0%), believed otherwise, while 32 (10.3%) admitted uncertainty.

**Figure 3:**
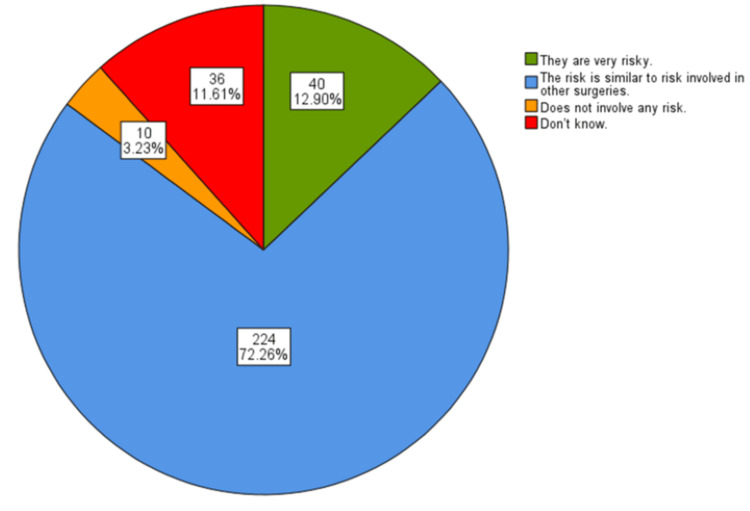
Participants' understanding of the risk involved in surgeries

Figure 3 is a pie chart showing participants' understanding of the risks involved in surgeries, focusing on cosmetic procedures. The majority of respondents, 224 (72.3%), perceived the risk as comparable to that of other types of surgeries, indicating a balanced understanding of surgical risks, while 40 (12.9%) considered these surgeries to be very risky. A small percentage, 10 (3.2%), believed these procedures involved no risk. Thirty-six (11.6%) participants expressed uncertainty.

## Discussion

Despite the rapid growth of plastic surgery in the past few decades, it remains poorly understood and underappreciated by the general public in Oman [10]. As far as we know, no research has been done regarding medical students' awareness of plastic surgery in Oman. Therefore, our study explored the knowledge and perceptions of plastic surgery among medical students at SQU. The study results showed a high level of awareness and the potential influence of media and clinical exposure on their educational and career considerations. Similar results were observed in a recent study among undergraduate male medical students in Saudi Arabia, which showed that 68% reported good knowledge regarding plastic surgery [6]. Moreover, despite reporting a high level of awareness in this study, 26 participants (8.4%) of the total sample had conclusively selected plastic surgery as a career. Conversely, a more significant percentage of participants, comprising 157 individuals (50.6%), reported a preference for other specialties outside of plastic surgery. In addition to those who have made firm choices, a significant percentage of participants, 127 (41.0%), remain undecided or undetermined about their specialty preference. Similar findings were observed in a study done in Saudi Arabia, where a large portion of Saudi medical students rejected plastic surgery due to cultural factors, despite acknowledging the importance of plastic surgery among them [6]. The results of this research revealed that more than half of the study sample, 174 (56.1%), reported exposure to both medically themed television and surgical disciplines. This pattern demonstrates that exposure to both medically themed television and surgical disciplines may have an equivalent impact on participants' considerations regarding specialty choice. Nevertheless, the primary and most frequently cited source of information influencing participants' knowledge and perceptions of plastic surgery in this study was the internet, with 217 (70%) participants indicating it as a key influence. Instagram was also a significant platform, influencing 182 (58.7%) participants, while friends were mentioned by 118 (38.0%) as a source of information. Traditional media, such as television, played a role for 97 (31.3%) individuals. Compared to the study by Fayi et al., recommendations from friends or family were the primary source of information, followed by social media [6]. However, other studies showed that television played a noticeable role in shaping their idea about plastic surgery [7,8]. Moreover, the previous study showed that television shows and advertisements influenced a relatively high number of students, reaching up to 34.2% [6]. Surprisingly, friends-gathering and discussions were the primary source of information for 62.8% of students, according to Gould et al. [8]. 

The study results revealed that age seems to be the most influential factor in participants' decision-making in plastic surgery, followed by educational level and GPA. Age showed a strong positive correlation (r = 0.758) with a highly significant p-value (<0.001), indicating that age is a significant factor affecting participants' decisions. The educational level had a weak positive correlation (r = 0.190) but remained statistically significant (p < 0.001), suggesting a minor influence on decision-making. In contrast, the academic GPA showed a moderate positive correlation (r = 0.310); however, the lack of a p-value suggests that further analysis is needed to determine its statistical significance, and a larger sample would provide more potent associations.

In the current research, 194 (62.58%) participants correctly identified cosmetic surgery as a subset of plastic surgery, demonstrating a solid understanding of the relationship between the two fields. However, 65 (20.97%) respondents did not share this view, and 32 (10.32%) were unsure, indicating some misconceptions or gaps in knowledge. Overall, several students know the conditions treated and procedures associated with plastic surgery. The current study indicates a significant interest among participants in both aesthetic enhancements and reconstructive surgeries. The most commonly recognized conditions included rhinoplasty (nose jobs) (224 students, or 72.3%), breast reduction or enhancement surgeries (205 students, or 66.1%), cleft lip and palate (197 students, or 63.5%), eyelid tears and facial cuts (197 students, or 63.5%), burns (193 students, or 62.3%), and congenital anomalies of the ear and nose (186 students, or 60.0%). Cosmetic procedures such as Botox (192 students, or 61.9%), abdominoplasty (tummy tuck) (134 students, or 43.2%), and liposuction (129 students, or 41.6%) were also frequently noted. Less commonly identified conditions included fractures of the hand (59 students, or 19.0%), diabetic foot wounds (51 students, or 16.5%), and tendon injuries of the hand (41 students, or 13.2%). Additionally, hair transplantation (114 students, or 36.8%), fractures of the jaw and face (143 students, or 46.1%), and reconstructive procedures like breast reconstruction post-cancer (196 students, or 63.2%) were also prevalent. These results are consistent with the findings of Agarwal et al. [11]. Plastic surgery was chosen most frequently for rhinoplasty, breast reconstruction, eyelid surgery, cleft lip surgery, and face lifting and tightening surgery, and less frequently for hand/peripheral nerve surgery and wound surgery [6,11]. However, according to a study by Wayne State University/Detroit Medical Center in the United States, medical students showed a gap in knowledge concerning plastic surgery and its relation to different subspecialties [7]. It is also important to highlight that the current study showed that 224 (72.26%) students viewed the risks associated with cosmetic surgery as comparable to those of other surgical procedures, reflecting a generally informed perspective. In contrast, 40 (12.90%) perceived these surgeries as highly risky, while 10 (3.23%) believed they carried no risk, and 36 (11.61%) were uncertain. These findings highlight varying levels of awareness and attitudes toward the nature and risks of cosmetic surgery among participants.

Limitations

This study has several limitations that future research should aim to address. First, the sample consisted solely of medical students from a single medical school in Oman, which limits the generalizability of the findings to all medical students in the country. Second, we did not evaluate the extent of students' exposure to or training in their respective career paths, which could introduce variability, even among students from the same medical school (e.g., participation in international exchanges or elective programs in plastic surgery). Finally, the findings were based on self-reported responses, which may be subject to bias.

## Conclusions

While SQU medical students demonstrated moderate awareness of plastic surgery, gaps in knowledge remain. Increased exposure through curricula and clinical rotations could enhance understanding and interest in this specialty, addressing misconceptions and improving patient care outcomes.
